# Malaria or kalimbe: how to choose?

**DOI:** 10.1186/1475-2875-8-280

**Published:** 2009-12-04

**Authors:** Bernard Carme

**Affiliations:** 1 Centre d'Investigation Clinique Epidémiologie Clinique Antilles Guyane CIC-EC 802, Cayenne General Hospital, Cayenne, French Guiana; 2 Laboratoire Hospitalo-Universitaire de Parasitologie et Mycologie Médicale, Equipe EA3593, UFR Médecine - Université des Antilles et de la Guyane, Cayenne, French Guiana

## Abstract

Should the Kalimbe (a traditional Amerindian loincloth) be banned, based on its association with an increased risk of malaria? Studies on malaria conducted on Amerindian children in the Oyapock region, French Guiana suggest that there is an argument for replacing the Kalimbe with a modern alternative. However, the wider issue of how the positive (risk reduction and related benefits) and negative effects (exacerbation of acculturation processes and associated consequences) should be assessed needs to be considered before suggesting a change in ancestral behaviour for medical purposes. A multidisciplinary approach is needed, together with caution and humility from epidemiologists.

## Introduction

The preservation of cultural practices is a fundamental, valid and attainable goal. However, whether ancestral customs should still be practised is brought into question when they go against hygiene education and disease prevention. Indeed, the introduction of hygiene and disease prevention measures is major factor contributing to the process of acculturation. In most cases, traditional customs are considered undesirable. For example, the prevention of neonatal tetanus requires umbilical cord asepsis, which is incompatible with Angolan traditional plasters made out of charcoal, palm oil and plant leaves [1]. However, more complex or delicate situations may arise.

**Figure 1:**
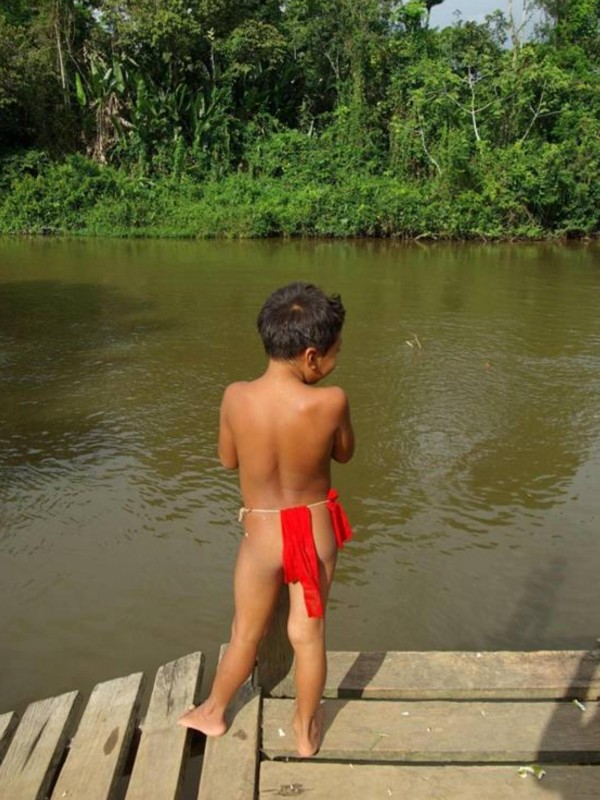
**Yayampi children wearing a Kalimbe (traditional Amerindian loincloth) in the village of Camopi on French Guiana's eastern border (Oyapock region)**.

In the Amazon, a region of endemic malaria, leishmaniasis and Chagas disease, people wearing a Kalimbe (a traditional Amerindian loincloth, figure 1) are more prone to insect bites than subjects wearing more protective clothing, and are therefore more exposed to the risk of vector-borne infectious diseases. This may seem obvious but evidence is still needed for a rigorous epidemiological investigation.

## Discussion

A recent observational study identified several factors that increased the risk of malaria in Amerindian children in French Guiana. The study was conducted in 2006 on children 0-5 years old living in the village of Camopi (Wayampi and Emerillon Amerindians) on the eastern border of French Guiana (Oyapock region). It was based on medical, environmental and behavioural predictive factors for malaria, using the Kaplan Meier method and Cox modelling [2].

Cox modelling identified five variables linked to a first attack of malaria (table 1). Clearing vegetation around the home was strongly correlated with a lower risk of contracting malaria, after adjusting for confounding factors. Also, residents of homes located more than 20 meters away from the river were at lower risk than those living less than 20 meters away. Over-crowding (children living in homes occupied by more than 6 people were at greater risk) continued to be associated with risk of malaria, even after adjustment for other environmental and behavioural factors, with Emerillon children being at higher risk than Wayampi children. Modern clothing appeared to be more protective than the Kalimbe.

**Table 1:** Risk factors of first malaria attack in Amerindian children [2]

		Hazard Ratio	95%CI	p
Vegetation around the home: % cleared	< 50%	1		
	50--75%	0.62	0.43--0.88	0.008
	> 75%	0.5	0.31--0.81	0.005
Distance from the home to the river	< 20 m	1		
	20--40 m	0.56	0.37--0.85	0.006
	40--80 m	0.72	0.47--1.09	0.128
	80--120 m	0.52	0.28--0.94	0.033
	> 120 m	0.5	0.30--0.86	0.012
Home: number of occupants	≤ 6	1		
	7	1.54	0.98--2.44	0.061
	8--11	1.9	1.29--2.81	0.001
	> 11	2.03	1.27--3.23	0.003
Ethnicity	Emerillon	1		
	Wayampi	0.552	0.401--0.796	< 0.001
**Clothing**	Kalimbe	1		
	Modern	0.64	0.46--0.90	0.011

Results for the multivariate Cox model, stratified for year of birth (saturated model with 23 variables which were significant at p > 0.25 in univariate analysis. The most parsimonious model was selected manually using log-likelyhood ratio test). Automated forward and backward stepwise elimination were consistent with the manual procedure above. Analysis time: 563.68 person-years. Proportional hazards assumption was checked (Schoenfeld: p = 0.862)

It is advisable to clear vegetation around homes to reduce the risk of malaria in children. However, in an unchanging Amazonian environment, recommending the removal of carbets (traditional Amerindian huts) away from the river is debateable, because the river represents a source of life, hygiene, transportation and entertainment. Furthermore, it is problematic to blame the Kalimbe for malaria at a time when Amerindian populations are faced with the loss of cultural identity with undeniably negative consequences. Loss of ancestral values (e.g. initiation rites based on courage, resistance to pain, respect for the environment and traditional activities such as hunting, fishing, slash and burn culture) without constructive alternatives is even more regrettable when some of these values have protected against established negative effects of behaviours such as alcoholism, consumerism, delinquency and rural exodus without the appropriate skills for living in the city.

Studies on clothing and exposure to vector-borne diseases have so far been limited to those involving military uniforms and clothing impregnated with insecticide [3,4]). However, one study addressed the effect of insecticide-impregnated and non-impregnated Islamic veils (more protective than the Kalimbe) on the frequency of insect bites related to malaria and leishmaniasis [5]. This use of insecticide-impregnated Islamic veils demonstrates a case in which traditional custom and the prevention of vector-borne diseases complement each other.

In general, changing ancestral behaviour because of links to risk factors should only be suggested after careful thought and justification, taking into account the potential negative consequences and risk of acculturation. The following points should be considered:

1) There should be no bias in the analysis model and a valid causal relationship should be demonstrated before questioning traditions.

2) Is the change for medical purposes only (morbidity, mortality of a number of cases, complications, deaths prevented per case and per year)?

3) Are there also economic aspects involved (amount of money saved)?

4) Can the benefits of programmed change be evaluated in terms of medical and economic factors?

5) What is the ratio of expected benefit to actual gain?

We thus need to consider not only the means used, but also the potential negative effects of the changes to be introduced and on the strength of tradition. Although the means can be quantified, the potential negative effects, and in particular, the strength of tradition, are more difficult to evaluate. Is it possible to place a value on ancestral traditions?

## Conclusion

The socio-cultural and philosophical dimensions of this issue are complex; and taking these factors into account does not simplify the methods of evaluation. Cultural globalisation seems to have its limits.

The way forward undoubtedly requires a multidisciplinary approach involving work in applied public health that requires cooperation with social and behavioural scientists and both caution and humility from epidemiologists.

## Competing interests

The author declares that they have no competing interests.
